# A vaccine to prevent cervical cancer: academic and industrial collaboration and a Lasker award

**DOI:** 10.1002/cti2.1002

**Published:** 2017-12-28

**Authors:** Edward M Scolnick

**Affiliations:** ^1^ Broad Institute of Harvard and Massachusetts Institute of Technology Cambridge MA USA

I joined the Department of Virus and Cell Biology at Merck Research Laboratories in 1982, when Dr Maurice Hilleman was head of the Department, and Dr Roy Vagelos was President of Research. At the time, there were lingering concerns about theoretical safety issues of Merck's first hepatitis B vaccine, called ‘Heptavax’, produced by purifying the native coat protein from the blood of virus‐infected patients. In the first few months of my tenure at Merck, I became aware of the effort in the Virus and Cell Biology Department to produce a vaccine against hepatitis B from the recombinant viral coat protein to alleviate these concerns. Merck had licensed an expression system from Chiron, a biotechnology company, that had developed a protocol for expressing the gene encoding this 25 kD molecular weight protein in yeast, and Merck was attempting to increase the expression level, purify the protein and formulate it into a vaccine. Unexpectedly, when the protein was purified from the yeast, it was poorly immunogenic, making it unsuitable for a vaccine. We attempted multiple purification methods, and, ultimately, the research team and I deduced and proved that, by using potassium thiocyanate, we could convert the monomer to a particle that resembled the particle of the native virus.^1^ Building on this discovery, Merck went on to produce the first vaccine made by recombinant DNA methods, and ‘Recombivax HB’ was approved in the United States by the FDA in 1986.

**Figure 1:**
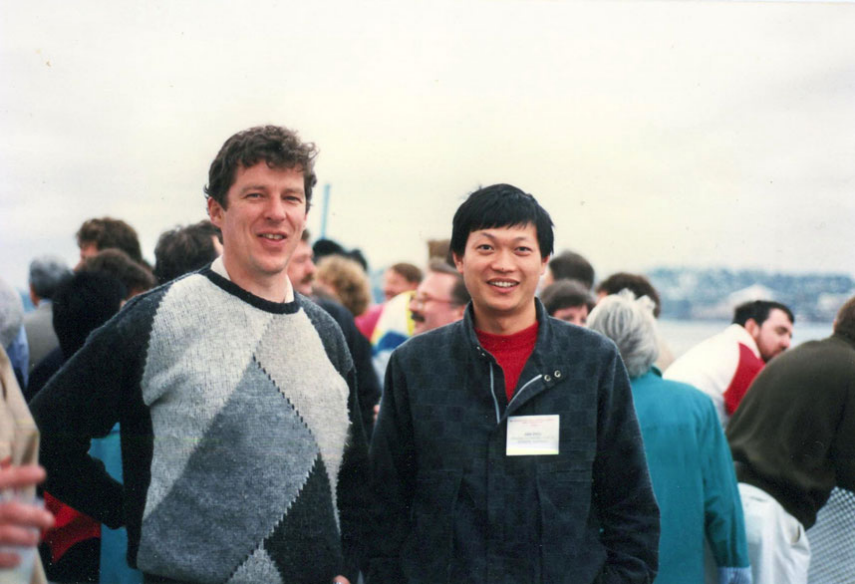
Ian Frazer and Jian Zhou in Seattle, at the HPV international conference (image supplied by Ian).

A few years passed, and in 1991, Jian Zhou and Ian Frazer, two virologists at the University of Queensland in Australia (Figure 1), stunned the audience at an international meeting on papillomaviruses (PVs) in Seattle. They presented data showing that they could produce, in mammalian cells using recombinant DNA methods, just the coat proteins (L1 and L2) of human papillomavirus 16, and the proteins assembled themselves into a virus‐like particle, without the DNA of the virus. They called this structure a virus‐like particle (‘VLP’). The audience was both stunned and sceptical of the work, but no less than Harald Zur Hausen, the Nobel Laureate who had discovered the role of HPV in certain human cancers, labelled the discovery as a breakthrough and a step towards a vaccine against cancer‐causing HPV genotypes.^2^ Many scientists who subsequently entered the vaccine field were at the meeting, including Drs Denise Galloway, Ricjard Schelgel, and Schiller and Lowy. It is noteworthy that the work begun in the National Cancer Institute was not begun until September 1991 as shown in the materials examined during the patent discovery process.

Shortly after this public meeting, a small group of scientists came to see me to ask for resources to begin a project to make a vaccine against HPV. They pointed out Merck's history of using the yeast expression system and purification technology in the hepatitis B vaccine project and argued that they could now do the same with HPV, after obtaining any needed licences to patents from the Australian group. I had mixed thoughts about this proposal. On the one hand, I was excited about the prospect of a vaccine to prevent certain cancers. On the other hand, I knew of the failures to show that a herpes simplex virus vaccine could prevent that sexually transmitted disease.^2^ Even during the HPV programme at Merck, other herpes simplex virus vaccines failed in clinical trials despite encouraging animal model data.^2^

Despite the uncertainties, after studying the biology of herpes simplex virus and HPV for a week, I met again with the Merck team and approved the beginning of the project. My decision was based upon the apparent fact that the mode of infection of herpes simplex virus was different from the mode of infection of HPV. Herpes was thought to infect both epithelial cells and superficial dendritic cells of the vagina, while HPV infected only epithelial cells, suggesting that the entrance to the latter might be blocked by a high‐titre serum IgG antibody and the assumption that enough would leak into the vagina to neutralise the HPV. There was no certainty that this approach would work and no certainty that we could induce a titre of IgG by systemic immunisation high enough so that some would leak into the vagina and neutralise the HPV virus. No animal model would increase the certainty, since herpes simplex virus vaccines had shown protection in animal models after systemic immunisation but, as noted, failed in human clinical trials.^3^ The medical need was great, the team led by Dr Kathrin Jansen was superb in my estimation, and I was therefore willing to take the risk and approved the start of the project.

The details of how the superb Merck team expressed the coat protein of four strains of the HPV virus, in yeast, how they did research to aid the reassembly of the monomer L1 protein into a stable VLP, and the meticulous protocols for clinical trials are all detailed in a review written by Bryan *et al*.^4^ I will not cover those here. I will point out that, in the way the trials were designed, the Merck team proved that the vaccine prevented not only the infection by the relevant HPV strains but also the development of a precancerous epithelial lesion induced by HPV. The details of how this was done are also described in the Bryan *et al*.^4^ review. Despite the prior work of Zur Hausen, there remained doubt in the field that HPV was actually the causative agent for cervical cancer. Since the vaccine prevented the precancerous lesion as well as the infection, the Merck trials leading to the approval of Gardasil convinced the scientific world.

Clearly, this project generated significant excitement at Merck and required many research innovations as detailed in Bryan *et al*.^4^ However, it also required the essential discovery that VLPs could be produced without the viral DNA. This was a crowded field. In some sense, all this work built upon an observation made on polyomavirus in 1986 by Salunke *et al*.^5^, who showed that the VP1 capsid protein of the murine polyomavirus expressed in *Escherichia coli* could self‐assemble into a viral capsid. However, production of the capsid required somewhat tricky *in vitro* manipulations. In contrast, for HPV, the VLP assembly could occur spontaneously in monkey kidney or insect cells infected with appropriate expression vectors and, subsequently, with after expression in yeast.^4^

For production of VLPs for HPV, many groups followed the Seattle presentation and published work of Zhou *et al*.^6^ In their first publication in *Virology*, Zhou and Frazer described the co‐expression of both L1 and L2 proteins and the production of VLPs. They used a strain of HPV brought from the United Kingdom by Zhou when he joined the Frazer laboratory. In their subsequent work, they produced HPV6 and 11 VLPs by expression of only L1.^6^, ^7^, ^8^ Many others reproduced the work. Rose *et al*.^9^ expressed the HPV 11 L1 protein, and Kirnbauer *et al*.^10^, ^11^ expressed the L1 protein of BPV1 and HPV16, showing effective production of HPV16 VLPs only once a mutation in the HPV16 L1 sequence of Gissmann^12^ had been corrected.^13^ Hagensee *et al*.^14^ expressed both L1 alone and L1 and L2 of HPV16. All of these papers expressed the proteins that formed HPV16 VLPs from the 2nd ATG of the HPV16 virus L1 gene, as taught by the Zhou and Frazer presentation, papers and patents. Thus, all the word after the Seattle presentation by Zhou and Frazer reproduced the observation that after expression in cells the coat protein(s) could self‐assemble into VLPs.

In addition to making HPV and animal PV VLPs, academic groups showed that these were immunogenic in animal models and that the immunogens protected canines^15^ or cottontail rabbits.^16^ Two groups, one academic and one industrial, showed immunogenicity in humans.^17^, ^18^ This was encouraging for the development of a HPV vaccine, although it was still a high uncertainty project because of the clinical failures of herpes simplex virus vaccines. Despite this uncertainty, Merck continued with its project to test an HPV vaccine for *efficacy* in humans, and continued in its effort to obtain appropriate licences from the academic group(s) that held the dominant patents. In a field as crowded as this, obtaining the licence turned out to be a significant problem.

There were many patents filed: Frazer, Schlegel, Schiller and Lowy, and Rose. Merck had to navigate the crowded patent literature to determine from whom to obtain licences. What were these patents?

1. On 19 July 1991, provisional filing by Frazer claimed the expression of both L1 and L2 using the second ATG in the HPV16 viral genome. In this patent, the claim was that both L1 and L2 were necessary and sufficient to produce VLPs. In the completed patent application on 20 July 1992, L1 was claimed to be all that was needed to produce HPV6, 11 and BPV1 VLPs. The earlier presentation by Zhou in Seattle and the paper noted above were cited in the patent. As noted above, this work was done on a strain of HPV that Zhou has brought to the Frazer laboratory. The concept discovered by Zhou and Frazer was clear. This patent application was published on 4th February 1993 as WO93/02184.
2. A patent application based on work led by Dr Douglas Lowy and Dr John Schiller at the National Cancer Institute was filed on 3 September 1992 and claimed VLPs using what was later shown by them to be prototype rather than wild‐type HPV16. The L1 protein was expressed from the second ATG as presented by Zhou in Seattle in 1991. In their subsequent filing of March 1993, they claimed they used a wild‐type HPV 16 virus sequence and that this, in contrast to the prototype sequence available in the literature, was able to assemble efficiently into VLPs. This patent application was published on 16 March 1994 as WO94/05792.

There was a contention in one of Lowy's patents that the actual virus sequence used by Frazer *et al*. had a mutant sequence at nucleotide 6241 that led to a histidine rather than an aspartic acid and that this mutation would have prevented a VLP from forming, as they had found themselves in their original paper and patent. In a letter to Lancet in 2006,^19^ Frazer and Cox pointed out that the virus (Zhou and Frazer) used for their recombinant vaccinia expression system was in fact a wild‐type virus and not a mutant with an inappropriate amino acid. This was unambiguously confirmed by sequencing the L1 gene in the recombinant vaccinia viruses deposited with the ATCC in April 1992 by Zhou and Frazer. The data for this were verified by independent sequencing also by lawyers that represented the Lowy patent to the USPTO. The patent was then awarded by the USPTO to the Schlegel group. The Frazer attorneys appealed this decision to the Court of Appeals of the Federal Circuit [CAFC] which mandated the USPTO to retract this decision and resulted in the granting of the dominant patent to the Australian group.

Merck (1992) and Glaxo Smith Kline (2004) took licences to the Australian patent, which subsequently became valuable because of this final patent ruling. Merck, and subsequently Glaxo Smith Kline, registered their HPV vaccines in the United States. In 2006, Merck registered a four‐valent vaccine containing HPV types 6, 11, 16 and 18, and Glaxo a two‐valent vaccine containing HPV 16 and 18 in 2009. The Merck vaccine was called Gardasil, and the Glaxo Smith Kline vaccine was called Cervarix. More recently, for reasons unknown to me, the Glaxo vaccine has been withdrawn from the US market, while in 2014, Merck introduced a nine‐valent HPV vaccine containing genotypes HPV 6, 11, 16, 18, 31, 33, 45, 52 and 58. Clearly, both the Merck and Glaxo vaccines have been a tremendous achievement for human health, preventing infection by HPV genotypes that cause cancer and precancerous changes in the vaginal epithelium. It is not an overstatement to contend that millions of lives will be saved by prevention of cervical and other cancers caused by HPV vaccines.

Following from this wonderful success story, in September 2017, a Lasker Prize was awarded for the work that led to this achievement. The prize was awarded to Lowy and Schiller. The contribution of Frazer was noted in the announcement. Schiller was quoted in a NY Times article on 6 September by Heather Murphy to say: ‘It is classic example, where we did something that companies weren't doing because it was too risky’. Clearly, the Merck programme began well before anyone proved in humans that such a vaccine would prevent infection, and well before Schiller published any of the vaccine development work from the NCI group. Thus, in my opinion, that statement is obviously inaccurate. Secondly, it is difficult for me to understand, given the history of the talk in Seattle, papers, patents and disclosures during patent interferences, and the final patent decision by patent Court that opines on patent claims that Ian Frazer was not a bona fide Lasker awardee. I can speculate that there was confusion about which strains of virus were used by which groups based upon publications. However, the Frazer and Cox letter to Lancet noted above and the ultimate patent decision should, in my opinion, have brought clarity to the field and to the committee. Many groups, including the NCI group, as noted above did very important work in this field, and they all deserve enormous credit for their contributions. Simply put, however, in my opinion, the Lasker committee made a puzzling decision, and Ian Frazer should have been a Lasker awardee because of his original seminal and correct contributions towards the generation of the HPV vaccine. Sadly, Dr Zhou had passed away. Perhaps award committees can learn from this case as they ponder recipients of other prestigious awards.

Production of a nine‐valent vaccine against HPV infection, which is estimated to protect against 90% of cervical cancer, 90% of genital warts and 90% of vulvar cancer, shows the power of collaborative academic and industrial research. Academic scientists anywhere in the world can make a basic research breakthrough discovery. Pioneering academic research in Australia and follow‐up work in the United States on VLPs, combined with pioneering research and creative and meticulous research and development in industry, has resulted in an amazing success in preventive medicine. I shout out a KUDO to all who helped this happen in both academia and industry. In an era of threats to research funding in academic centres, perhaps this case study can be a paradigm of how novel and important preventive medicines and treatments can be brought forth for human benefit.
